# A shift in focus: Mothers’ descriptions of sharing a child with a co-parent with unhealthy alcohol use after participating in a support program

**DOI:** 10.1186/s13722-023-00369-y

**Published:** 2023-02-15

**Authors:** Ola Siljeholm, Veronica Ekström

**Affiliations:** 1 Centre for Psychiatry Research, Department of Clinical Neuroscience, Karolinska Institutet, & Stockholm Health Care Services, Region Stockholm Riddargatan 1, 114 35 Stockholm, Sweden; 2 Stockholm Centre for Dependency Disorders, Stockholm Health Care Services, Region Stockholm, Stockholm, Sweden; 3 Department of Social Sciences, Marie Cederschiöld University College, Sköndal, Sweden

**Keywords:** Conventional content analysis, Cognitive behavioral treatment, Qualitative research, Concerned significant other, Harms to others, Parental alcohol problems, Web-based intervention

## Abstract

**Background:**

Unhealthy alcohol use (UAU) affects not only the drinking individual, but also significant others (SOs), such as partners and children. Most of the harm to others caused by alcohol can be attributed to common, moderate drinking patterns, but existing studies have mainly included SOs of individuals with severe UAU. There is a need for increased knowledge regarding SOs of individuals in an earlier stage of UAU and efficacious support programs for this group. The aims of this study were to investigate reasons for seeking support as described by SOs sharing a child with a co-parent with UAU and to investigate how SOs perceived effects of a web-based self-delivered support program.

**Methods:**

A qualitative design conducting semi-structured interviews with 13 female SOs sharing a child with a co-parent with UAU. The SOs were recruited from a randomized controlled trial of the web-based program and had completed at least two of four modules in the program. Transcribed interviews were analyzed using conventional qualitative content analysis.

**Results:**

Regarding reasons for seeking support, we created four categories and two subcategories. Main reasons were wanting validation/emotional support and coping strategies for handling the co-parent, and negative perceptions of available support options for SOs. Regarding perceived effects of the program, we created three categories and three subcategories. Main effects were an improved relationship to their children, increased own positive activities, and less adaptation to the co-parent, though SOs also mentioned what was perceived as missing in the program. We argue that the interviewees represent a population of SOs living with co-parents with slightly less severe UAU than previous studies and therefore provide new insights for future interventions.

**Conclusions:**

The web-based approach with potential anonymity was important for facilitating support-seeking. Support for the SOs themselves and coping strategies for co-parent alcohol consumption were more common reasons for seeking help than worry about the children. For many SOs, the program was a first step in seeking further support. Spending more dedicated time with their children and being validated as living under stressful conditions were described by the SOs as particularly helpful.

*Trial registration* The trial was pre-registered at isrctn.com, reference number ISRCTN38702517, November 28, 2017

## Background

In Sweden, approximately 15% of men and 12% of women show patterns of unhealthy alcohol use (UAU) [1]. UAU refers to risk drinking as defined in the Alcohol Use Disorders Identification Test-Consumption [2, 3], and/or consequences as described in the criteria for harmful use in the International Classification of Diseases, 10th revision (WHO, 2010). For every individual with UAU, at least one person—but likely more—is negatively affected by their drinking [4, 5].

Life as a significant other (SO) to a person with UAU is associated with elevated levels of psychiatric conditions such as substance use disorders, depression, and trauma, but also somatic problems and impaired quality of life [6, 7]. The risks of negative consequences increase with proximity to the person with UAU [5] and women are more affected than men [8].

Research on female SOs sharing a child with a co-parent with UAU has shown high levels of stress and strain and that all aspects of life are negatively affected [8–10]. Other studies describe female SOs experiencing multiple burdens, such as caring for both the co-parent and children, being responsible for the household, trying to compensate for the unreliable and absent father, and feeling powerless [4, 11] and that they need support to deal with the challenges they face [10].

Children growing up with at least one parent with UAU have increased risks for several negative psychiatric and social consequences [12–16]. Although this is a vulnerable group of children, very few take part in any kind of support intervention, mainly because the population is hard to reach [17]. There is substantial evidence that a parent who does not have UAU can function as a protective factor for children in families where the other parent drinks too much [9, 16, 18].

There is evidence that family-oriented interventions ameliorate substance use problems and improve family functioning and SO mental health [19, 20]. There is also growing evidence for integrating substance use treatment and parental training for individuals with substance use disorders [21]. No previous trials have investigated integrated approaches focusing on SOs and children with a co-parent/parent with UAU, and there is a need to develop programs that could improve SO health and possibly prevent the development of risk factors.

The present qualitative study is part of a larger project where a web-based, self-delivered support program for SOs sharing a child with a co-parent with UAU was developed and evaluated in a randomized controlled trial (RCT), reported in [22]. The program comprised core components from Community Reinforcement Approach and Family Training (CRAFT) [23, 24] and a Swedish parenting training program called All Children in Focus (ACF) [25]. Both CRAFT and ACF are manualized interventions based on the principles of behavioral therapy. CRAFT has been shown to improve SO mental health in several trials, e.g., [26, 27], and to increase the treatment entry rate for the relative with alcohol or substance use problems [28]. In CRAFT, SOs practice behavioral strategies with three main goals: (1) to improve quality of life; (2) to decrease the substance use of the relative by minimizing positive consequences of substance use and increasing positive reinforcement of sober activities and; (3) to increase the relative’s motivation for treatment. However, CRAFT does not include strategies for handling children exposed to parental UAU. ACF provides parents with communication skills, tools to promote positive child behaviors and cease to promote negative behaviors, and information on how to prevent and handle conflicts, rule-setting, and boundaries [25, 29].

The web-based self-delivered design was intended to make the program accessible to SOs across Sweden and to enable anonymity for SOs. This was done to reduce barriers, since this is a group unwilling to seek support due to stigma, shame, or fear of revealing alcohol problems in the family to authorities [30, 31]. A study protocol with further details on the RCT has been published [32].

The specific aim of the present study was twofold: (i) to investigate reasons for seeking support as described by the SOs; (ii) to investigate how the SOs perceived the effect that the program had on themselves, the children, and the drinking co-parent.

Findings from this study could contribute to new insights regarding how to reach and help SOs at an earlier stage of the development of UAU in a co-parent, knowledge most likely not limited to the specific situation of SOs sharing a child with a co-parent, but applicable to SOs in general.

## Methods

### The SPARE intervention

The program Supportive PArenting and REinforcement (SPARE) consisted of four modules (displayed in Table 1). All modules were divided into three themes: (a) Enhance SO’s quality of life; (b) Strategies for understanding and handling the co-parent with UAU; (c) Parenting strategies.

**Table 1:** SPARE content and exercises

Content	Exercises
1 a) Introduction and information. Set goal for program use b) Decreasing ineffective strategies in trying to change the co-parent’s alcohol consumption. Safety precautions c) Strategies for dedicated parent–child time	Make room for own positive activities Practice 15 min daily of dedicated parent–child time
2 a) Strategies for SOs to enhance own well-being b) Mapping patterns of co-parent alcohol consumption; triggers, behaviors, and short/long-term effects (functional analysis) c) Talking about alcohol with children. Give positive attention to appreciated child behaviors	Set a personal goal for own well-being Mapping drinking situations Focus on appreciated child behaviors 15 min daily dedicated parent–child time
3 a) Increase SO self-respect through cognitive exercises and rewards b) Five positive communication skills. Mapping and analyzing interplay with the co-parent c) Mapping situations of parental and child conflicts. Increase positive child behaviors	Continue working on goal for own well-being Practice positive communication and mapping interactions with co-parent 15 min daily dedicated parent–child time
4 a) Handling negative emotions. Where to find more support for SO or child b) Encourage help-seeking in co-parent. Let co-parent handle negative consequences of drinking c) Handling conflict situations with children. Rules and boundaries; making agreements with children about responsibilities	Planning ahead (maintaining own changed behaviors over time, supporting co-parent positive behavior change over time, preparing for possible setbacks) Continued dedicated parent–child time

### Design of the present qualitative study

The present study had a qualitative design and we conducted interviews with SOs who had completed at least two of the four modules in SPARE. For other eligibility criteria, see [22].

We used a semi-structured interview guide with open-ended questions. The interview started with the broad question *Tell me about yourself and your family and why you applied to the study*. Other examples of central questions included: *How has the program affected you as a parent?* and *In what way has the program affected your understanding of your co-parent’s relationship to alcohol?*

After completing the 12-week follow-up assessment (see [22]), we approached SOs via e-mail. Thirteen of 42 SOs expressed interest to participate and were interviewed.

The characteristics of the interviewed SOs are presented in Table 2. All 13 SOs were women who shared at least one biological child aged 3–11 years with a male co-parent.

**Table 2:** SO characteristics

SO	Age (years)	Relationship to co-parent	Number of affected children	Number of years with perceived UAU for co-parent
1	50	Ex-partner	2	10
2	35	Partner	2	4
3	44	Ex-partner	1	15
4	41	Ex-partner	1	6
5	44	Partner	2	10
6	44	Partner	2	20
7	36	Partner	3	7
8	46	Partner	3	3
9	42	Ex-partner	2	5
10	37	Partner	2	6
11	44	Partner	4	1
12	34	Partner	4	5
13	44	Partner	2	6

We conducted all interviews in 2019–2020, either face-to-face, via videoconference, or by telephone. The interviews lasted approximately 45–60 min. We recorded all interviews and they were subsequently transcribed verbatim by a professional transcriber. We sent written information to all SOs in advance, and read through the study information together with the SOs before commencing the interviews.

We analyzed the transcripts using conventional qualitative content analysis (hereafter “conventional content analysis”) as described by Hsieh and Shannon [33]. We used an inductive approach to code the data without a pre-defined code system or analytical framework. The coding in conventional content analysis results in categories and subcategories which aim to elucidate structures and relationships in the phenomenon under study. We deemed this inductive categorization suitable considering the limited amount of previous research available regarding SOs who share children with a drinking co-parent and their experiences of need for support.

First, we read the transcribed interviews from beginning to end several times. Next, we read the texts more thoroughly to identify codes that were present in the text, or create codes consisting of a few words to encapsulate the meaning of a certain sentence. Within each content area, we investigated the relationships between the codes and jointly discussed and formulated suggested categories and subcategories.

Quotes are presented in the results section to increase the transparency of the analysis and facilitate assessment of the study’s credibility and transferability.

## Results

We divided the results into two content areas, as suggested by [34], with categories and subcategories. These are presented in Table 3.

**Table 3:** Content areas, categories, and subcategories

Content area	Reasons for seeking support	Perceived effects of the program
Categories and sub-categories	Coping with co-parent drinking - Escalation of drinking and alcohol-related consequences - Wanting new coping strategies in relation to the co-parent Emotional validation and support Worrying about the children Perceptions of available support for SOs	Change in my own behavior - Interaction with the children - Taking care of myself - Coping with the co-parent Acknowledgement and relief Missing in the program

## Reasons for seeking support

### Coping with co-parent drinking

#### Escalation of drinking and alcohol-related consequences

Most of the interviewed SOs did not state that a particular event triggered their support-seeking behavior, but rather a continuous escalation of consequences related to their co-parent’s alcohol consumption, or feeling so emotionally affected that it was impossible to ignore. In many of the SOs’ descriptions, becoming parents changed the way they perceived the co-parent’s alcohol consumption.

*We both, like, enjoyed partying and stuff, like you do when you are a student […] but after only a year or so I started noticing that he had, like, he didn’t drink in the same way as me. […] Then we had children and [his consumption] kind of kept chafing in me. *(5)

Something that recurred in several of the interviews were how the SOs’ own boundaries and tolerance of co-parent alcohol-related behaviors had shifted significantly over the years, slowly and almost imperceptibly.

*If it would go from one day to the next, you would say that this isn’t ok, but since it’s always sneaking up on you, you push your boundaries forward all the time for what is okay. *(1)

#### Wanting new coping strategies in relation to the co-parent

Although the SOs were aware of all the things they were putting up with, most of them had decided to stay in the relationship with their co-parent, for various reasons. They expressed how their partner, when sober, was a fantastic parent and spouse, but that his alcohol consumption made him unreliable, dull, absent, selfish, and, in some cases, mean. They all had experiences of arguing and fighting with the co-parent, which in some instances could make things better for a while—but the co-parent always returned to his previous behaviors over time. Hence, many SOs were looking for new strategies to deal with the co-parent’s alcohol consumption because all their previous attempts had failed.

### Emotional validation and support for myself

Several SOs mentioned wanting to compare their own circumstances to those of others, to see if what they experienced was common or not. Many of the SOs had asked themselves “is this normal?”, “am I overreacting?” or “should I put up with this?”. They described how the co-parent’s alcohol consumption had caused them to feel hurt and betrayed and that the situation had eventually led to a general feeling of distress and uneasiness.

*Yes, I noticed feeling bad, I understood that I needed help in some way as well, and that I would, perhaps, I don’t know, but I needed help to feel a little better, since I didn’t have anyone to talk to […]. *(8)

Several SOs described feeling shame due to the social stigma associated with alcohol problems in the family. However, the experience of keeping the secret of the co-parent’s alcohol problems and the related consequences to oneself eventually became consuming, which led to them seeking support.

### Worry about the children

All of the SOs expressed concern and worries regarding how the co-parent’s alcohol consumption might affect the children in the longer term, or had at least started to wonder *if* it might affect them.

*It’s not like he consciously mistreats them in any way, I really don’t think that, but he, kind of, doesn’t connect the negative sides and it takes a copious amount of space and perhaps other people come second, yes. *(9)

Some of the SOs explicitly stated that they were searching for strategies for and knowledge on handling the effects that the co-parent’s alcohol consumption had on their children. For example, one SO described what she hoped to gain from the program:

*Yes, some keys to what happens in a child when it is disappointed in a parent and what it could, what consequences it could have, you know. I mean, some help with how to help him handle it, that is really what I want. *(4)

In some cases, the SOs said the main reason for staying in the relationship was that they worried about how the children would be treated if they separated and they did not want the children to live alone with the co-parent every other week. However, most SOs described a more general worry that their children might be affected in the future.

### Perceptions of available support for SOs

The final category in the interviews was related to difficulties for the SOs to find support that they perceived as appropriate. In Sweden, the social services in the municipalities are responsible for providing support. Most of the SOs in this study were aware of this, but did not perceive this as an appealing alternative. Some SOs had tried to contact social services, but were either told that their partner had to seek help first or were unable to find support. Many SOs described being reluctant to contact social services, out of fear of possible repercussions, such as that their children would be placed in foster care. Although this is very rare and only occurs in cases of major neglect or threats to the children’s safety, several SOs mentioned this as a reason.

In small municipalities where everybody knows where the social services are, SOs feared that others in the community would find out if they went there. Hence, for several SOs, the possibility of anonymity offered in the SPARE program was appreciated.

*I live in a small town, I work within health care, I kind of felt like I didn’t want to contact anyone, because it becomes so obvious, it’s always people you know, working in the same field. So, I thought, well maybe there is something on the internet, so I started to Google and found this. […] An anonymous tool like this was very valuable. *[2]

The knowledge and experiences of self-help groups and non-profit organizations varied among the interviewed SOs. Some mentioned being aware of Al-Anon groups, but not being interested in attending meetings, either for practical reasons or due to the religious content. SOs residing in larger cities were aware of a few non-profit organizations, something that SOs in other parts of Sweden did not have as an option, which several of them mentioned.

Many SOs stated that the online delivery of the program suited their needs, due to either geographical issues, time-planning reasons or the potential for anonymity. Several mentioned that they did not perceive their situation as urgent enough to seek help in the health care system or social services and they felt that the study could provide an appropriate level of support, or serve as a first step.

## Perceived effects of the program

### Acknowledgement and relief

Practically all the SOs described one of the most evident effects of the program being a sense of relief at being acknowledged in that the circumstances they were living under were not normal. Many SOs had doubted themselves and their perception of the co-parent’s drinking as causing stress and strain on both them and their children. Many co-parents had told the SOs that they exaggerated the negative effects of their consumption. The program, as well as learning about others’ experiences, helped SOs realize that they were entitled to being angry or stressed and that they were in a situation that justified seeking support.

*Because you get called crazy a lot when living with such a person, a lot of things become normalized. So, when you asked these questions, it was a kind of acknowledgement that I’ve been living with a man with a disease. *(3)

Several SOs described how the emotional validation they got from the program made them calmer, which allowed them to be more present in the now and helped them shift focus to more positive aspects of life. Many also appreciated writing free-text responses in the program mentioning that it had had a relieving and almost therapeutic effect.

### Change in my own behavior

Empowered by the program, several SOs felt entitled to confront the co-parent, to talk more openly to the children, and to make room for activities of their own. Hence, acknowledgement played a crucial role in enabling the SOs to start making changes.

#### Interaction with the children

A description recurring in virtually all the interviews was that the SOs started to spend more time with their children, as an effect of the program. The program recommended 15 min of dedicated parent–child time per day, which most of the SOs practiced regularly during the program, though perhaps not as frequently after its end. Several SOs described that even if they did not set time aside daily for the children anymore, an awareness of the importance of being present and giving the children more attention was established as an effect of the program.

Many SOs reported talking more to the children, having more fun with the children again, and experiencing fewer conflicts with them. One SO gave this answer to the question what she had gotten from the program:

*Really a lot, especially in my role as a parent. [The children] get a little, you know, I wouldn’t say “forgotten,” but you focus so much on other things with thoughts and everything. And there were really good exercises and thoughts, like this thing that you are supposed to take ten minutes with them and things like that. […] I think it was really good. *(12)

#### Taking care of myself

After participating in the program, most of the SOs mentioned having become better at taking time for themselves and prioritizing doing healthy activities for their own sake. In many cases, physical activity was mentioned as especially helpful for regaining energy, leading them to feel calmer and better prepared for dealing with daily chores and interactions with the children, as well as with any situations arising from the co-parent’s drinking.

Another aspect of taking care of themselves was shifting focus away from trying to influence the co-parent when he was drinking—leaving him alone at such times and instead doing an activity on their own. This was considered to conserve a lot of energy that was otherwise spent on negativity and arguments.

#### Coping with the co-parent

Several SOs mentioned that they felt their capacity for maintaining clearer boundaries for themselves had improved, meaning that they no longer adapted as much to co-parent alcohol-related behaviors. This shift in SO behavior resulted for example in co-parents having to deal with the negative consequences of their alcohol consumption on their own.

*Before, […] if we had decided to go somewhere on a Sunday to do something, if he had been drinking and was tired, then I probably would have been like “ok, but I’ll wait then.” I would have delayed it and left at 12 o’clock instead of at ten or so. But now I leave at ten o’clock no matter if he comes along or not, and I guess that has made him get it together most of the time and he has joined us. *(2)

Some of the SOs could clearly describe having made changes in their behaviors towards the co-parent, though with differing results. The most prominent behavior change appeared to be a shift from complaining about the co-parent’s shortcomings towards using a more positive tone when communicating.

*Like, “What a nice weekend we’ve had” instead of, like, “How nice that you didn’t get hammered today.” You know what I mean, really tried to talk to him in another way and I hope that has had a positive effect on him. *(7)

Some SOs had perceived changes in the co-parent’s alcohol consumption which they could relate directly to a change in their own behavior. One SO described an occasion when her co-parent was deeply asleep when he was supposed to work. Previously she would have stayed at home and tried to wake him, but this time she took the children to her in-laws and told them the reason for her leaving the children there. This led to an argument with the co-parent which resulted in him not drinking for a month afterwards.

### Missing in the program

For some SOs, participating in SPARE was not perceived as enough; they described more or less a status quo regarding the co-parent’s drinking and having a need for additional support. No one said they had gained *nothing* from participating in the program, maybe just not what they had hoped for. This was especially true regarding communication with the co-parent, understanding their motives for drinking, and seeing effects on the co-parent’s alcohol consumption. Several SOs described having difficulties applying the communication strategies in their daily life—only one SO stated explicitly that it had been useful to her.

The most common perception among the SOs of what was missing from the program was interaction with a living person. One SO recounted asking the staff a question and getting a response that was validating:

*It felt like, “oh, I want to reply again so I could get another answer,” it felt very nice. Just that little, kind of, real contact […] not just an automated response, “thank you for handing in your first assignment,” but perhaps there could have been a sentence so you saw that someone had read it. *(5)

However, the same SO also said that the current online approach made her express herself more openly and honestly compared with when she had previously met a therapist face-to-face, so it was not clear to her what kind of approach was best.

A majority of the SOs said that they would like additional support after completing the program, but the need was less than when they entered the study. The need for support was greatest in connection to setbacks in the co-parent’s drinking. Some SOs had already sought further support via non-profit organizations, social services, or private alternatives and said that participation in the study was an important first step in the process.

## Discussion

In this study, we interviewed 13 female SOs sharing a child with a male co-parent with UAU after they had participated in a web-based self-delivered support program called SPARE.

We found that the main reasons for seeking support were connected to the SOs’ own emotional needs deriving from co-parent alcohol consumption and trying to understand or cope with consequences of the co-parent’s drinking. These findings are in line with the widely used Stress–Strain-Coping-Support (SSCS) model [35]. The model has been developed after decades of studies on SOs in various cultures. Common themes described by SOs who are seeking support are for example threats to the SOs and the family, difficulties with coping strategies, barriers to receiving support, and increased levels of own mental health problems [4, 8]. The reasons described by the SOs in our study are coherent with the previous research which shows that the SSCS model is applicable to SOs with differing levels of accumulated burdens. 

The results are also consistent with those of a qualitative study in Australia analyzing themes for help-seeking in online counselling transcripts of partners contacting a national web-based service for substance and alcohol use [36]. The study found three broad themes with seven sub-themes, including Seeking advice, Wanting to talk, Discussing help-seeking, and Coping processes. The authors concluded that web-based counselling was important in facilitating support-seeking for SOs, since it lowered the barriers and could be a first step to seek further support, if it was insufficient in itself—a conclusion that is supported by the results of our current study. Further, most participants in our study described being reluctant to contact social services. This is in line with the results of a study on barriers and facilitators regarding SO help-seeking by McCann et al. [30], which showed that previous negative experiences of authorities, self-stigma, and public stigma were barriers. We argue that the anonymity provided in SPARE was a key aspect in facilitating for SOs to enter the support program.

The gender asymmetry in our sample reflected the RCT, where 96% of the participants were female, a proportion similar to those in all previous CRAFT trials, with women constituting 72–100% of the participants [28]. Studies from several different cultures confirm that the burden on female SOs is greater than that on male SOs due multiple responsibilities such as taking care of both children and husband or risk of intimate partner violence [8, 37]. Additionally, sharing a child often comes with greater strains on women [11, 38]. A co-parent with UAU adds a burden to the women taking care of the family. We interpreted the SOs’ statements about unreliable, dull, absent, selfish, and mean partners, and the constant low-key stress that at times erupted in major stressful events, as descriptions of such strains. In essence, we suggest that Swedish female SOs also experience heavier burdens than male SOs, explaining at least part of the gender asymmetry.

Regarding perceived effects of the SPARE program, most SOs described feeling calmer and relieved, had started to put their own needs first, and adapted less to the co-parent. This is in line with the results of a study by Hellum et al. [39], where self-delivered CRAFT was one of three forms of program distribution (the others being face-to-face individual sessions and group sessions) and the written material was found to be helpful mainly in improving SOs’ quality of life. Hellum et al. discuss the importance of SOs being met in a non-judgmental and accepting manner to increase awareness of their not being alone and feeling acknowledged as living under stressful circumstances. We draw the same conclusions from the interviews in our study.

Although almost none of the SOs mentioned looking specifically for parenting strategies when searching for support, almost everyone described how one of the main appreciated effects of SPARE was related to an increased focus on their children. After setting aside 15 min daily for dedicated parent–child time, the SOs described fewer conflicts with the children, having more fun together, and talking more openly to each other. We argue that a new insight from this study is how the 15-min strategy can be applied in a situation where the main problem is not children with disruptive behaviors or a malfunctioning parent–child relationship, but where one parent’s shortcomings (in this case UAU) lead to neglect and other forms of negative consequences for the children. Dedicated parent–child time seemed to help the SOs shift focus away from arguments with the co-parent, towards the children. The positive effects on the relationship with the children were clearly described in several interviews. The protective factor of a good relationship with one parent when the other parent has substance use problems is well-established [9, 16, 18] and we suggest that the present study shows that one of the main effects of SPARE is an improved parent–child relationship. Hence, we would propose that support programs for SOs should incorporate this kind of parental intervention to improve circumstances for affected children.

Although most SOs described feeling concern about how the children might be affected, the consequences of the co-parent’s consumption were not perceived as severe enough to cause the children serious harm. This result can be compared to a previous Swedish qualitative study [40] where 23 (female) SOs sharing a child with a (male) co-parent with UAU were interviewed. In that study, all but two of the SOs had separated from their co-parents and stated that the reasons for this were severe substance use and major parental neglect, involvement of social services, and children showing delinquent behaviors. The 23 SOs all identified as parents to children who fared poorly as a consequence of co-parent drinking, a characteristic that the SOs in our study did not seem to share. Based on this, we reason that the consequences of co-parent UAU must be rather severe for SOs to initiate treatment-seeking *based primarily on wanting support for affected children*. In the present study, a majority of the SOs were still in a relationship with their co-parent, and their descriptions of perceived needs focused mainly on their own well-being and strategies for dealing with or relating to the co-parent. This is considered important knowledge for the development of support programs that can attract SOs at an earlier stage of co-parent UAU.

Lastly, several SOs mentioned that they felt personalized feedback was missing. This desire for feedback is understandable, but must be related to the potential gains of offering a program that is available at all times and does not require staffing, which for some SOs can be enough—at least as an important first step.

### Strengths and limitations

A strength of this study is that it reached a population that, to our knowledge, has not previously been represented in the literature. Thus, the results presented here are important for future studies aiming to attract this population. However, it is possible that the decision to include only participants who completed *two or more* of the modules in the SPARE program led to a risk of survival bias in our results.

One limitation is that the sample of SOs in our study was ethnically homogenous, with a high level of education, and thus does not reflect the Swedish population. However, we consider the current sample representative of the group of SOs who participated in the RCT and believe the results of the interviews are transferable to the larger study population.

An aspect regarding credibility involves the authors’ perspectives and experiences. The first author is a clinical psychologist specialized in cognitive behavior therapy and was involved in all parts of the RCT. The second author has a background in social work and was not involved in the quantitative evaluation. The close involvement of the first author comes with a risk of bias, potentially leading to more positive interpretations of data than by a “neutral” observer. This risk was highlighted already from the start of the study and we tried to counteract it by continuously discussing the analyses and results back and forth between the authors.

## Conclusions

The SOs confirmed that offering a web-based program with possible anonymity was a successful strategy for facilitating help-seeking. The main reasons for seeking support were wanting emotional validation/support and coping strategies in relation to the co-parent. The perceived effects were related mainly to improved own well-being thanks to feeling acknowledged, an increase in positive, healthy behaviors, an improved relationship with the affected children, and giving less attention to co-parent drinking. The self-delivered design was appreciated, though several SOs missed having a person provide personalized feedback. Though the perceived effects of SPARE were not enough for some of the SOs to feel that they no longer needed any support, it was an important first step for them to seek further support. Feeling validated as living under stressful circumstances and spending more dedicated time with their children appeared to be particularly helpful to the SOs.

## Data Availability

In order to protect the participants’ identities, the qualitative data analyzed in the current study have not been made publicly available. The transcribed interviews are in Swedish. Translated pseudonymized data are available from the corresponding author on reasonable request.

## Abbreviations

UAU: Unhealthy alcohol use
SO: Significant other
CRAFT: Community Reinforcement Approach and Family Training
ACF: All Children in Focus
SPARE: Supportive PArenting and REinforcement
RCT: Randomized controlled trial
SSCS-model: Stress–Strain-Coping-Support-model
